# A Diagnostic and Management Challenge in Severe Fatal Mercury Poisoning Secondary to Homeopathic Medications

**DOI:** 10.7759/cureus.86514

**Published:** 2025-06-22

**Authors:** Harry Edgar-Whelan, Eleanor Fish, Phyoe Kyaw Pyae, Hakim Ghani, Rama Vancheeswaran

**Affiliations:** 1 Acute Medicine, Watford General Hospital, Watford, GBR; 2 Acute Medicine, University College London Medical School, London, GBR; 3 Respiratory Medicine, Royal Papworth Hospital, Cambridge, GBR; 4 Respiratory Medicine, Oxford University Hospitals NHS Foundation Trust, Oxford, GBR

**Keywords:** adult nephrotic syndrome, ayurvedic medicine, homeopathic medicine, mercury poisoning, nephropathy

## Abstract

We present a case of a patient known to have rheumatoid arthritis with severe nephrotic syndrome very likely due to chronic mercury poisoning. She was known to have rheumatoid arthritis for 10 years. Mercury poisoning was attributed to homeopathic Ayurvedic medicine which she took for arthritic pain. It is highly likely that the Ayurvedic medications contained high concentrations of mercury. The patient presented with worsening breathlessness on exertion and global pitting oedema. Initial investigations suggested a nephrotic syndrome of unknown aetiology. The patient’s kidney function worsened, and she developed a hospital-acquired infection and deteriorated further with type 1 respiratory failure. Despite attempted continuous veno-venous hemodiafiltration, the patient unfortunately passed away secondary to end-stage renal failure caused by chronic severe mercury poisoning. This case report adds to the concerns regarding the potential risks of unregulated Ayurvedic medications without clear instructions from a trained medical professional. It highlights the importance of taking a detailed drug history on the first encounter with the patient.

## Introduction

Mercury in all forms - elemental, organic and inorganic - is known to be hazardous to humans if consumed [1,2]. Chronic mercury exposure is common in populations who consume high levels of fish; however, severe mercury poisoning remains rare [3]. Mercury poisoning can manifest in non-specific, multisystem symptoms depending on the form of mercury driving the pathology. Ingested mercury often causes a wide range of non-specific symptoms involving gastrointestinal disturbance including pain and nausea. It can also cause renal dysfunction due to toxic nephropathy [4]. Ayurvedic medications are herbal-based medications mainly found and used in the Indian subcontinent. These medications are derived from plants but can include animal products and heavy metals [5].

We report a case of unexpected severe mercury poisoning in the United Kingdom (UK), secondary to herbal Ayurvedic medicine. This manifested as nephrotic syndrome, progressing to end-stage renal failure and ultimately the unfortunate death of a young patient. This case report highlights a rare and deadly presentation that is underreported. In developed countries with an evolving demographic coupled with the rising popularity of “alternative medicine”, this is an important consequence to be aware of [6].

## Case presentation

A lady in her 40s was suffering from breathlessness, poor urine output and generalised oedema for several weeks. She presented to the emergency department with significant breathlessness, oliguria and anasarca.

**Figure 1:**
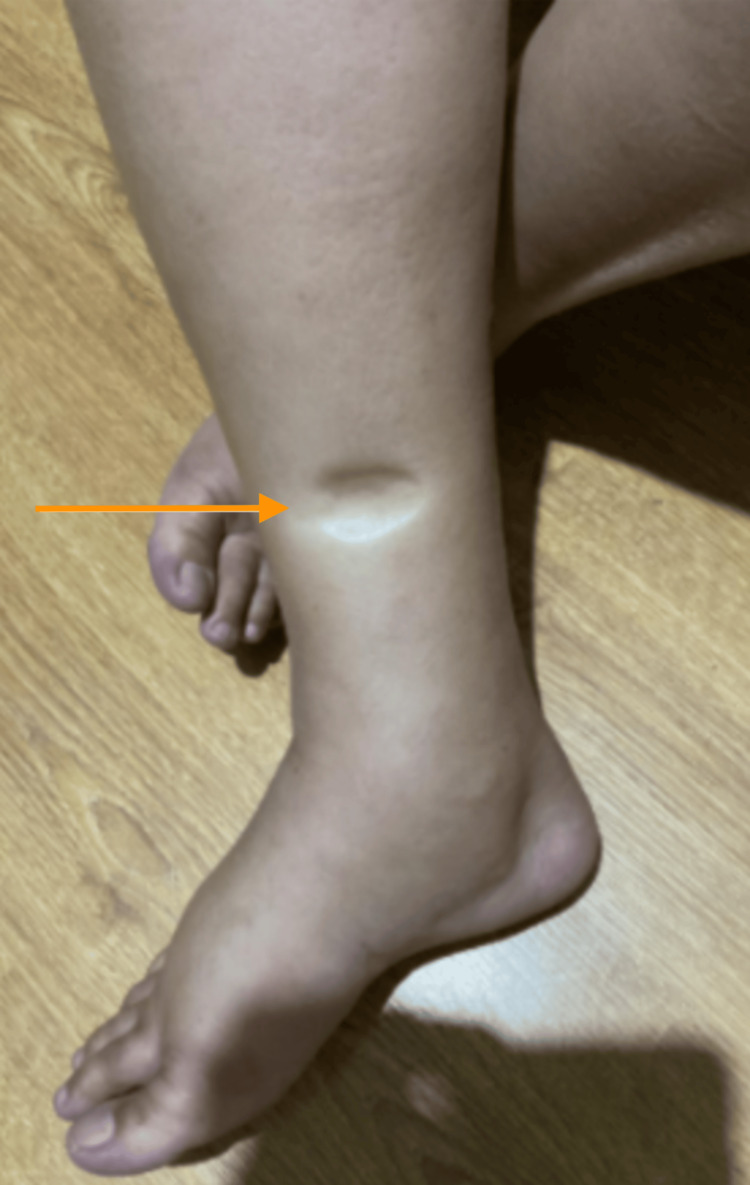
Pitting oedema in the lower right limb of a woman in her 40s. This extended to both upper limbs.

Clinical examination on presentation to the emergency department demonstrated significant pitting oedema in the lower limbs (Figure 1) extending up to the abdomen and upper limb, inability to complete full sentences, and minimal urine output. This was the first time she experienced these symptoms. Following review by the acute medical physician and preliminary investigations, she was admitted to the acute medical unit with suspected nephrotic syndrome.

The patient maintained a healthy lifestyle, did not smoke cigarettes or drink alcohol, and was not on regular prescribed medication. She did have potentially hazardous occupational exposure as a tailor. She voluntarily informed the attending medical physician of having a 10-year history of arthritis which she self-medicated with herbal Ayurvedic medicine. She consumed the traditional herbal medicine for two consecutive months but stopped two months before presentation to the hospital.

Investigations

Preliminary investigations included blood tests with full blood count, renal profile, bone profile, liver function test and C-reactive protein (CRP), which showed severe hypoalbuminemia, microcytic anaemia and hyperlipidaemia (Table 1). Thyroid function tests were requested to assess severe hypothyroidism as a cause for severe oedema and proteinuria and were in keeping with hypothyroidism [7,8]. Hypothyroidism was attributed to mercury interfering with the iodine uptake within the thyroid gland, reducing free thyroid hormone levels. Iron studies were done and were normal. She had a mildly elevated N-terminal pro B-type natriuretic peptide (NT-proBNP) on admission. Hypocalcaemia was secondary to hypoalbuminemia, resulting in a normal corrected calcium. Further tests were sent to determine the underlying aetiology of proteinuria [9,10].

**Table 1:** Blood test results on admission and day 4 of inpatient stay. eGFR: estimated glomerular filtration rate; T4: Thyroxine; HDL: High-density lipoprotein; LDL: Low-density lipoprotein; NT-ProBNP: B-type natriuretic peptide

Test Name	Results on Admission	Results on Day 4	Units	Reference Range
Full blood count				
White Cell Count	5.4	0.7	10⁹/L	3.2-10.5
Red Blood Cells	4.73	2.47	10¹²/L	3.8-5.8
Haemoglobin	108	59	g/dL	11.5-16.5
Haematocrit	0.33	0.17		0.315-0.44
Mean Cell Volume	70.7	70.5	fL	81-100
Platelets	227	53	10⁹/L	122-410
Urea and Electrolytes				
Sodium	135	137	mmol/L	133-146
Potassium	5	2.8	mmol/L	3.5-5.3
Urea	6.9	10.4	mmol/L	2.5-7.8
Creatinine	86	221	umol/L	45-84
eGFR	71	23	mL/min	>90
Liver Function Tests				
Alanine transaminase	7		U/L	0-39
Alkaline phosphatase	156		U/L	30-130
Total bilirubin	4		umol/L	0-20
Albumin	5	7	g/L	35-50
Total protein	40		g/L	60-80
Globulin	35		g/L	30-35
Thyroid Function Tests				
Thyroid Stimulating Hormone	8.73		mu/L	0.55-4.78
Free T4	12.8		pmol/L	9.5-22.7
Bone Profile				
Calcium	1.89		mmol/L	2.2-2.6
Adjusted Calcium	2.16		mmol/L	2.2-2.6
C-Reactive Protein	<5.0	200	mg/L	0-5
Lipid Tests				
Cholesterol	12.5		mmol/L	0-4.9
HDL Cholesterol	0.85		mmol/L	> 1.0
Triglyceride	3.75		mmol/L	< 1.7
LDL cholesterol	9.95		mmol/L	< 2.6
NT-ProBNP	345	14254	pg/mL	<100
Troponin	<5	170	ng/L	0-5

An autoimmune renal screen performed showed strongly positive antinuclear antibodies (ANA) and anti-Sjögren's-syndrome-related antigen A (SS-A/Ro60) which were in keeping with her history of rheumatoid arthritis rather than an alternative autoimmune pathology.

Urine dipstick test was strongly positive for protein and red blood cells, suggestive of glomerular pathology. 24-hour urine collection was started on the advice of a nephrologist. This quantitatively confirmed nephrotic syndrome. She produced 3.4 g/L of protein across 24 hours, grossly above the normal range of 0-0.13 g/L. Urine protein/creatinine ratio was 925.9 mg/mmol (normal value 0-15).

**Figure 2:**
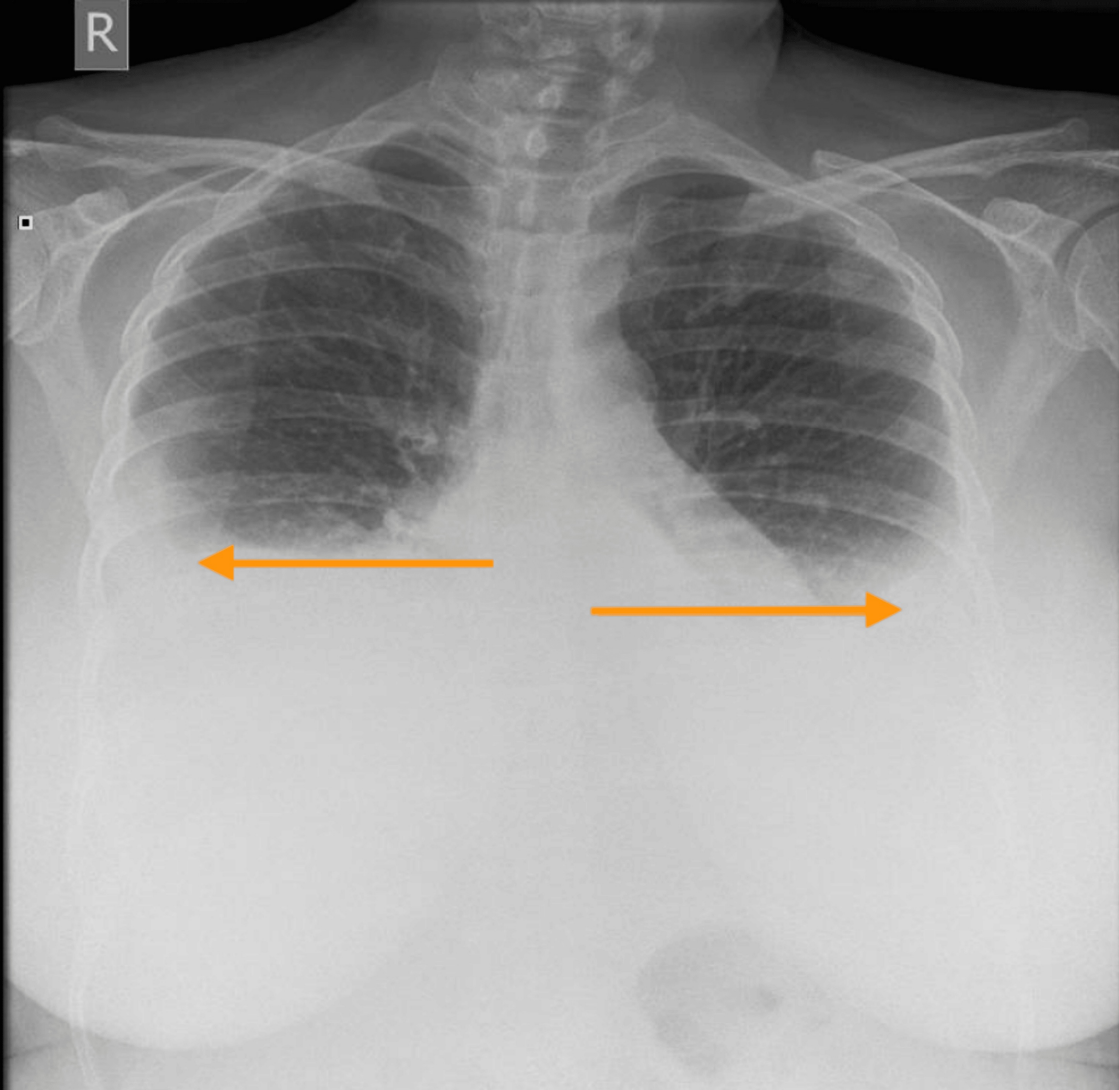
Admission chest X-ray. Bilateral pleural effusions delineated by arrows.

**Figure 3:**
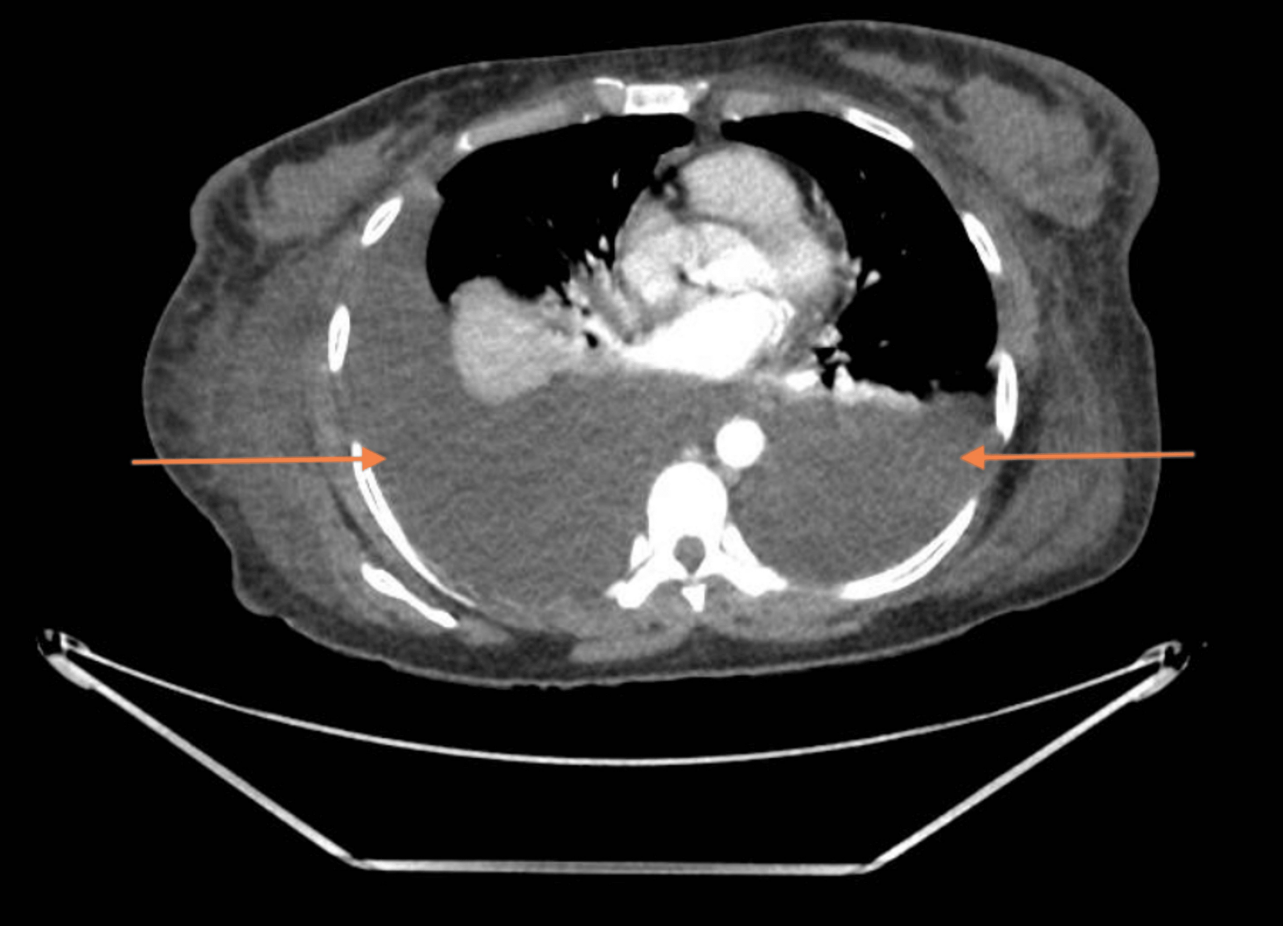
CT chest cross slice from CT chest abdomen and pelvis. Significant bilateral pleural effusions delineated by arrows.

**Figure 4:**
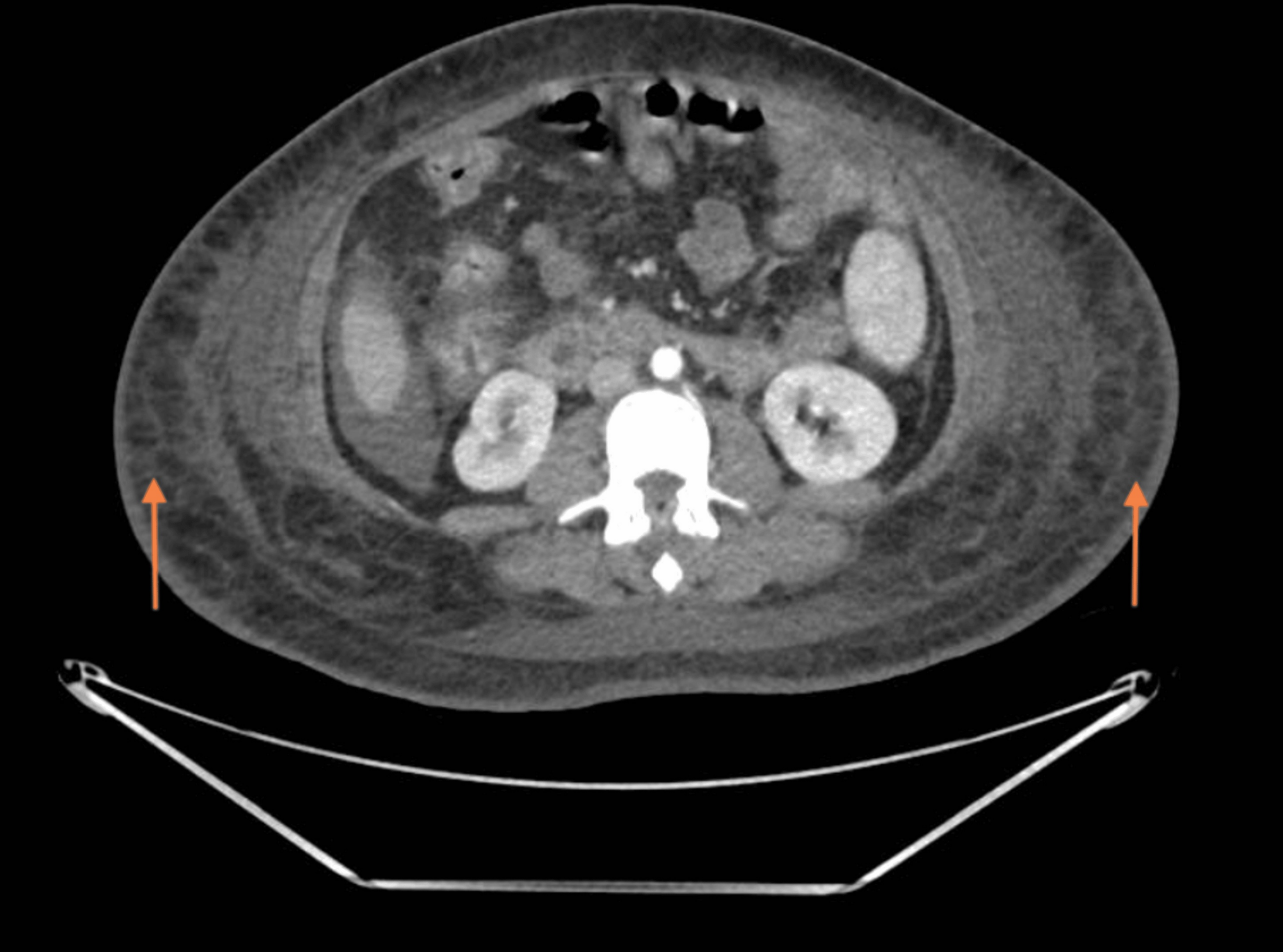
CT abdomen cross slice from CT chest abdomen and pelvis. Significant bilateral subcutaneous oedema delineated by arrows.

The chest X-ray (Figure 2) illustrated bilateral moderate pleural effusion but no other abnormality. Transthoracic echocardiography demonstrated normal biventricular sizes and function with no significant valvular disease. This was used to rule out a cardiogenic cause. Post-renal cause of renal impairment was excluded by ultrasound of the kidneys and urinary tract. A computed tomography (CT) scan of her chest, abdomen and pelvis was performed to screen for malignancy. The CT scan (Figures 3, 4) showed bilateral pleural effusion, ascites, generalised subcutaneous oedema and minimal cortical scarring of her right kidney. This suggested that there was neither a malignant nor an obstructive renal cause. We then had to consider other aetiologies, namely heavy metal poisoning.

Given the use of traditional herbal Ayurvedic medicine, a blood toxicology screen was obtained to ascertain any heavy metal poisoning [5,11], which was sent to an external laboratory.

During her hospital stay, the patient’s blood test results worsened markedly. She developed acute, significant pancytopenia, and her kidney function declined, necessitating urgent renal replacement therapy (Table 1). Additionally, elevated NT-proBNP and troponin levels suggested cardiac strain, while a sudden rise in CRP was attributed to an Extended-Spectrum Beta-Lactamase-producing (ESBL) Escherichia coli septicaemia.

In retrospect, these findings could have been due to bone marrow failure and end-stage renal disease due to chronic mercury poisoning [12,13]. As the patient clinically deteriorated, her blood gases showed worsening metabolic acidosis and type 1 respiratory failure.

Differential diagnoses

The patient presented with anasarca and breathlessness where initial investigations showed severe hypoalbuminemia, proteinuria and hyperlipidaemia. As nephrotic syndrome was a primary candidate for her presentation, primary and secondary glomerulonephritis were the top differential diagnoses [9,10]. As the patient’s onset of symptoms was rapid, it was more likely that she had glomerulonephritis caused by a rapidly progressive autoimmune disease or secondary to a toxic agent. Proliferative glomerulonephritis was less likely as she did not meet the criteria for nephritic syndrome. Congestive cardiac failure was considered but NT-proBNP level and echocardiography were non-concerning. We also considered secondary amyloidosis and cryoglobulinaemia vasculitis. We acknowledge there are cases where rheumatoid arthritis can induce a nephrotic syndrome, particularly when treated with older generations of disease-modifying anti-rheumatic drugs like cyclosporine [14]. However, in the absence of any additional nephrotoxic insult, this is highly unlikely.

Treatment

The patient received 240 mg of continuous furosemide infusion over 24 hours throughout her admission for diuresis as she was symptomatic with generalized oedema. Furosemide was initially beneficial as it helped manage breathlessness, but this was short-lived due to the inability to determine a reversible cause for severe proteinuria and decline in renal function which ultimately led to clinical deterioration. Low-dose levothyroxine was initiated for hypothyroidism discovered as part of her investigations.

As the patient had severe anaemia and hypoalbuminemia, she received a transfusion with two units of packed red blood cells and 100 ml of 20% human albumin solution. Meropenem and gentamicin were initiated for suspected hospital-acquired infection which later showed ESBL Escherichia coli cultured septicaemia. Sodium bicarbonate 8.4% was initiated to correct the acidosis.

She ultimately required intensive care unit (ICU) admission for inotropic and ventilation support, and continuous veno-venous hemodiafiltration (CVVHDF) for further deterioration of renal function with fluid overload and metabolic acidosis. A right-sided chest drain was inserted to purely ease ventilation as the effusion was found to be transudative. Throughout the hospital admission, the patient’s treatment had been targeted at managing her symptoms whilst waiting for investigation to determine a diagnosis, which proved to be elusive and challenging.

Outcome

**Figure 5:**
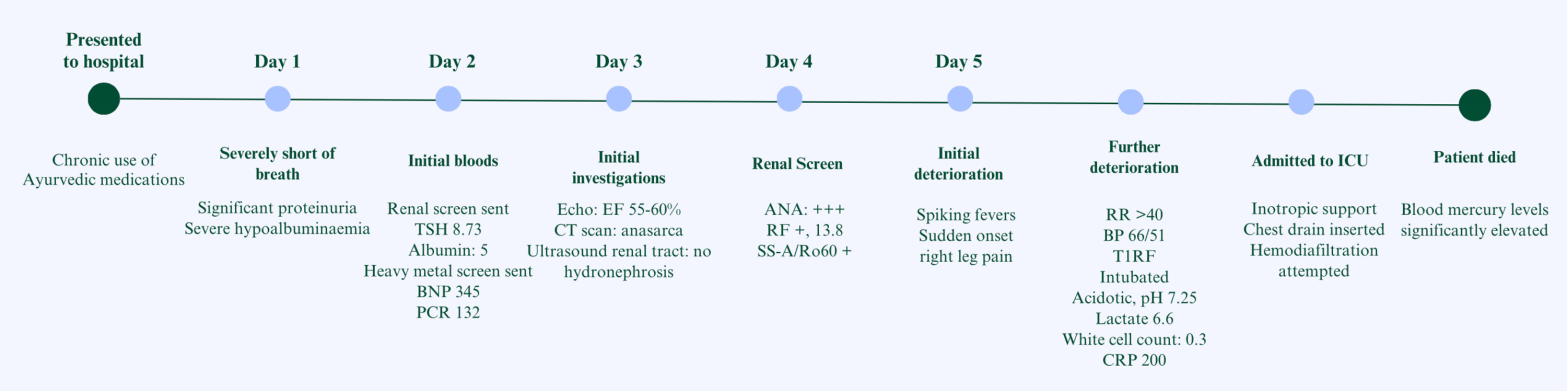
Summary timeline of events. TSH: Thyroid-stimulating hormone; BNP: Brain natriuretic peptide; PCR: Protein creatinine ratio; EF: Ejection fraction; ANA: Antinuclear antibody; RF: Rheumatoid factor; SS-A/Ro60: Sjögren's-syndrome-related antigen A; RR: Respiratory rate; BP: Blood pressure; T1RF: Type 1 respiratory failure.

This patient was reviewed by a nephrologist during her stay in the acute medical ward. The plan suggested continuation of diuresis and arranged plans to transfer her to a renal tertiary care centre. However, she suffered an acute deterioration with type 1 respiratory failure and worsening metabolic acidosis before transfer was completed. The patient’s clinical deterioration (Figure 5) was attributed to progressive kidney pathology and hospital-acquired infection resulting in sepsis, evident from a sharp rise in CRP and requiring inotropic support.

The patient required admission to the ICU where she was intubated and mechanically ventilated. CVVHDF was attempted but failed as the patient deteriorated even further. The patient unfortunately passed away later that day in the ICU, one week into her admission to the hospital. Results from the blood toxicology screen for heavy metals (Table 2) were only received after the patient’s death. The blood mercury level was over four times above the normal acceptable laboratory level [15]. The aetiology of her nephrotic syndrome was most likely due to severe mercury poisoning from her herbal Ayurvedic medicine.

**Table 2:** Results of heavy metal screen from admission.

Test Name	Results	Units	Reference range
Arsenic	13	nmol/L	14-95
Cadmium	7	nmol/L	0-27
Lead	0.97	nmol/L	0-0.24
Mercury	109	nmol/L	0-25

## Discussion

Mercury is a recognised neurotoxicant and immunotoxin, which has been designated by the World Health Organisation as one of the 10 chemicals of public health concern. The pathogenesis of mercury-induced kidney disease has not been fully elucidated, but evidence suggests a combination of dose-related tubular dysfunction likely secondary to membranous nephropathy with a role for an immune reaction [2,16].

Medical literature generally presents the more well-known neurological manifestations of mercury poisoning. There are few cases published regarding the impact of mercury poisoning on the kidneys, least so within the UK. The rising use of alternative medication calls for physicians to be aware of potentially toxic side effects. Doshi et al. [17] published a series of five cases linked to chronic mercury poisoning causing membranous nephropathy secondary to traditional Indian medicines, and hence should always be enquired about when assessing the drug history of a newly admitted patient.

There are no clear guidelines published on the medical treatment of mercury; however, the National Institute for Health and Care Excellence suggests supportive treatment until the mercury is cleared or using chelating agents including dimercaptosuccinic acid (DMSA) under guidance from the National Poisons Information Service.

The co-administration of furosemide and albumin in patients with nephrotic syndrome is debated in the literature. Currently, no randomised clinical trials have concluded a clear recommendation for treatment. The strongest recommendation for treatment is in patients with severe nephrotic syndrome who are established as diuretic resistant (showing no improvement in urine output and sodium excretion) should trial supportive transfusions of albumin aiming to shift fluid to the intravascular space [18]. This aims to aid diuresis and assist in the management of nephrotic syndrome. It was attempted in this patient, but ultimately did not yield a positive outcome.

With the current resources available to our trust, we were unable to obtain the heavy metal screen results prior to the patient’s death. This was exacerbated by the late presentation of the patient. The efficacy of chelation for symptomatic mercury intoxication decreases with the time interval since exposure [19]. Advice from the National Poisons Information Service should be sought and consideration of early empirical chelation therapy should be considered when diagnostic suspicion is high [19].

Of interest in this case is the source of the mercury poisoning. When taking a patient’s history, it is crucial to ask about the use of homeopathic medications, particularly those not regulated by governmental bodies. Homeopathic medicine use varies across Europe, with prevalence rates of 1% in the UK and Ireland and over 10% in France, Switzerland, and Germany [5,6]. As the market continues to grow, physicians must be aware of the potential risks associated with unregulated treatments. In the branch of Ayurvedic medicine of rasa shastra, metals are added for their perceived healing qualities [5]. We cannot comment on whether the medications were contaminated or deliberately contained high quantities of mercury. Of a 230-product sample of Ayurvedic medications in the US, 40.6% contained mercury concentrations ranging from 13-28 mg/g [20]. This is significantly above the FDA action level for dietary mercury in fish, which is 0.001 mg/g. Ayurvedic medications should be a significant concern in cases of suspected mercury poisoning.

## Conclusions

We suggest that nephrotic syndrome of unknown aetiology requires detailed medical and drug history including alternative medication which can be elusive unless directly addressed. Rapidly progressing nephrotic syndrome necessitates suspicion of heavy metal poisoning with confirmation through laboratory screen. Management of mercury toxicity involves supportive treatment and chelating agent dimercaptosuccinic acid (DMSA), but should be discussed with the National Poisons Information Service.

There are several case reports of accidental mercury poisoning in young children via inhalation or ingestion of broken thermometers; however, it can also present nefariously in adults which is rare in a developed country but should be considered due to evolving demographics and increased use of alternative medications.
